# Children’s Consumption Patterns and Their Parent’s Perception of a Healthy Diet

**DOI:** 10.3390/nu12082322

**Published:** 2020-08-03

**Authors:** Jessica Eliason, Francesco Acciai, Robin S. DeWeese, Sonia Vega-López, Punam Ohri-Vachaspati

**Affiliations:** College of Health Solutions, Arizona State University, Phoenix, AZ 85004, USA; jjeliaso@asu.edu (J.E.); rsdewees@asu.edu (R.S.D.); Sonia.Vega.Lopez@asu.edu (S.V.-L.); Punam.Ohri-Vachaspati@asu.edu (P.O.-V.)

**Keywords:** fruit consumption, vegetable consumption, added sugar intake, children and adolescents, dietary guidelines, parental perception of the healthfulness of their child’s diet

## Abstract

This study aims to examine children’s fruit, vegetable, and added sugar consumption relative to the Dietary Guidelines for Americans and the American Heart Association’s recommendations, as well as to compare children’s reported consumption with parental perception of the child’s overall diet quality. Data were drawn from 2 independent, cross sectional panels (2009–10 and 2014–15) of the New Jersey Child Health Study. The analytical sample included 2229 households located in five New Jersey cities. Daily consumption of fruit (cups), vegetables (cups), and added sugars (teaspoons) for all children (3–18 years old) were based on parent reports. Multivariate linear regression analyses estimated children’s adjusted fruit, vegetable, and added sugar consumption across parents’ perception categories (Disagree; Somewhat Agree; and Strongly Agree that their child eats healthy). Although only a small proportion of children meet recommendations, the majority of parents strongly agreed that their child ate healthy. Nonetheless, significant differences, in the expected direction, were observed in vegetable and fruit consumption (but not sugar) across parental perceptional categories for most age/sex groups. Dietary interventions tailored to parents should include specific quantity and serving-size information for fruit and vegetable recommendations, based on their child’s age/sex, and highlight sources of added sugar and their sugar content.

## 1. Introduction

Every five years the United States Department of Agriculture (USDA) and the United States Department of Health and Human Services (DHHS) update the Dietary Guidelines for Americans (DGA) based on current scientific evidence [1]. The USDA’s MyPlate is a platform for communicating information and recommendations in the DGA to the public to promote healthy eating [2]. Over the years these guidelines have consistently advocated for nutrient-dense diets that promote consumption of fruits and vegetables and limit foods and beverages with added sugars. Recommendations for fruits and vegetables vary based on children’s age and sex. For example, based on the 2015 guidelines, children ages 2–8 should consume 1–1.5 cups of fruit and 1–1.5 cups of vegetables, while children 9 and older should consume 1.5–2 cups of fruit and 2–3 cups of vegetables. The guidelines also recommend that all children, regardless of age and sex, limit their consumption of added sugars to less than 10% of daily calories. The American Heart Association (AHA) recommends that all children between 2 and 18 years of age limit their added sugar intake to less than 25 g (or 6 teaspoons) per day, regardless of the calories consumed [3].

Despite these recommendations, the vast majority of children continue to consume energy-dense, nutrient-poor diets. Vegetable consumption among children is particularly dismal, with only about 2–16 percent of children across all age groups meeting recommendations [4]. While young children (ages 2–5 years) usually consume adequate amounts of fruit [5], more than 75% of older children and adolescents fall short of the recommendations [4]. Although added sugar consumption has decreased for children across all age groups in recent years, the average intake still exceeds 10% of daily calories per day [4]. Sugar-sweetened beverages, including fruit drinks (e.g., juice) and soft drinks (e.g., soda) are the main sources of added sugars among children ages 2–18 [6]. Unsurprisingly, 12–18 year-olds have a higher intake of sugar-sweetened beverages than younger children [7,8]).

Increasing children’s fruit and vegetable intake and limiting added sugar consumption requires interventions in home and school settings [9]. Parents’ dietary consumption [10], modeling behaviors [11], and feeding styles [12] can influence children’s diets. Arguably, improving children’s consumption is also closely linked to how parents perceive the healthfulness of their child’s diet because a parent must first recognize when their child’s diet is poor or needs change in order to take actions [13,14]).

Briefel et al. (2015) used parent 24-h recalls to assess young (<4 years old) children’s dietary adherence to the American Academy of Pediatrics “5-2-1-0” recommendations (5 fruits and vegetables, 2 h of screen time, 1 h of physical activity, 0 sugary beverages), and examined parental perceptions of children’s diet quality by asking, “Do you think your child gets enough fruits and vegetables?” About three quarters of parents perceived that their child consumed enough fruits and vegetables, yet only 31% of these young children consumed the recommended 5 servings [14]. Similarly, Kourlaba and colleagues, in a cross-sectional analysis conducted in Greece, compared parental perceptions with preschoolers’ (2–5 years old) diet quality [15]. Parental perception was measured through the question, “How would you characterize your child’s diet?” with 5 ordinal response options ranging from ‘no good’ to ‘very good,’ while diet quality was measured using parent 24-h recalls and then quantified into a Healthy Eating Index (HEI) score. In this sample, 83% of mothers overestimated their preschooler’s diet quality. Based on HEI scores, 18% of the children had a poor diet, 81% had a diet that needed improvement, and only 0.2% had a ‘good’ diet. However, only 3% of mothers said their child’s diet was poor, 17% said their child’s diet needed improvement, and 80% of mothers considered their child’s diet healthy. No significant associations were found between parental overestimation and socio-demographic characteristics. To date, no studies have been conducted in the U.S. to compare parental perception and dietary intake across a broad childhood age spectrum.

This study fills an important gap in the literature by (1) examining 3–18-year-old children’s fruit, vegetable, and added sugar consumption relative to the DGA and (2) comparing these consumption patterns with parental perception of the child’s overall diet quality.

## 2. Materials and Methods

### 2.1. Participant Data

Data were drawn from the New Jersey Child Health Study (NJCHS), a longitudinal study examining the relationship between food and physical activity environments and children’s weight status and related behaviors. Data used for the current analysis were collected from two cross-sectional panels that used random digit phone surveys (landline and cell phone) in 2009–2010 and 2013–2014. Surveys were conducted in English and Spanish and included questions on demographics and dietary habits of an adult respondent and one randomly selected index child (3–18 years old). The survey respondent, referred to as parent hereafter, was the adult most knowledgeable about the household’s food shopping and, in over 94% cases, was either a parent or grandparent of the index child.

Data for panel 1 were collected in 2009–2010 from 1708 households across five cities in New Jersey: Camden, New Brunswick, Newark, Trenton, and Vineland. Data collection for panel 2 took place in 2013–2014 and included 803 households from four cities: Camden, New Brunswick, Newark, and Trenton. Oral consent was obtained prior to the start of the interview. The Rutgers and Arizona State University Institutional Review Boards approved the study.

### 2.2. Analytical Variables

The analytical sample (*n* = 2229) included households from panel 1 and panel 2 surveys with complete data. Similar to other studies [14,15,16], parents were asked to evaluate the diet quality of their children. Parents were asked to respond to the following statement: “In general, (Index Child) eats healthy” using the following response options: “Strongly agree,” “Somewhat agree,” “Somewhat disagree,” “Strongly disagree,” or “Don’t know and refused.” This analysis combined “Somewhat” and “Strongly” disagree into one category due to the small percentage (4%) of parents who strongly disagreed that their child ate a healthy diet. Children whose parents responded “don’t know” or refused to answer were excluded from this analysis (<0.5%).

Parents were also asked, in a separate section of the survey, to report on frequency of consumption of fruits, vegetables, and foods and beverages with added sugars for the index child. Parent-report of children’s and adolescents’ dietary intake has been found to be similar to child [17,18] or adolescent self-reports [18,19]. Because the current study includes younger children, for whom self-reports are not a viable instrument, parent reports of consumption were used for children of all ages (3–18 years old). This approach was used to ensure consistency of responses across age groups and households and is in line with previous research [20,21].

Frequencies of consumption of fruits and vegetables were derived using questions adapted from the Behavior Risk Factor Surveillance Survey and 2009–2010 National Health and Nutrition Examination Survey (NHANES) [22,23], with response options of number of times per day, week, or month. For fruit consumption, parents were asked (1) “Not counting juice, how often did (Index Child) eat fruit? Count fresh, frozen or canned.” (2) “How often did (Index Child) drink 100% pure fruit juices, such as orange, apple, or grape juice? Do not include fruit flavored drinks with added sugar like Hi-C, Gatorade, or fruit punch.” For vegetable consumption, a series of questions were asked: (1) “How often did (Index Child) eat a green leafy or lettuce salad, with or without other vegetables?” (2) “Not including French fries or other fried potatoes, how often did (Index Child) eat any other kind of potatoes, such as baked, boiled, mashed potatoes, or potato salad?” (3) “How often did (Index Child) eat cooked or canned dried beans, such as refried beans, baked beans, bean soup, tofu, or lentils?” (4) “How often did (Index Child) eat other vegetables, such as tomatoes, green beans, carrots, corn, cooked greens, sweet potatoes, broccoli, or any other kinds of vegetables?” For analysis, the four questions above were combined to create a total vegetable consumption variable.

Frequencies of consumption of food and beverages with added sugars were measured from the following questions, which were also adapted from national surveys [22,23]: (1) “How often did (Index Child) drink fruit flavored drinks, such as lemonade, Sunny Delight, Kool-Aid, Gatorade, or sweet iced teas? Do not include 100% fruit juice.” (2) “How often did (Index Child) drink regular carbonated soda or soft drinks that are sweetened, such as Coke, Pepsi, or 7-up? Do not include diet drinks.” (3) How often did (Index Child) eat sweet items, like cookies, cakes, candy, or pies?

Demographic data were obtained from parents’ responses to questions regarding parent’s and child’s sex, age, race/ethnicity, mother’s education, household income, and household participation in the Supplemental Nutrition Assistance Program (SNAP) and in the Special Nutrition Assistance Program for Women, Infants, and Children (WIC) in the previous 12 months. Because MyPlate translates DGA for various food groups into age- and sex-specific recommendations [2], children in this study were categorized into six age/sex categories accordingly: 2–3 year-olds, 4–8 year-olds, 9–13-year-old males, 9–13-year-old females, 14–18-year-old males, and 14–18-year-old females.

### 2.3. Statistical Analysis

Frequency of consumption of fruit, vegetables, and foods and beverages with added sugars were converted to per day values and then transformed into quantities; specifically, cups per day for fruits and vegetables, and teaspoons per day for added sugars. Conversion into quantities was based on the algorithm provided by NCI, which is based on the sex-age specific portion size estimate for each food from the NHANES 24-h recall data [24]. Fruit and vegetable recommendations in cups for specific age/sex groups were obtained from MyPlate guidelines. Because children’s daily caloric intake, required for estimating MyPlate-based added sugars recommendations, was not measured in our household surveys, we chose to use the American Heart Association’s (AHA) recommendation for children, which suggests consuming ≤25 g (or ≤6 teaspoons) of added sugar [3], regardless of age or sex.

All analyses were run in Stata [25]. Multivariate linear regression analyses were performed to estimate children’s fruit, vegetable and added sugar consumption by parents’ perception categories (Disagree; Somewhat Agree; and Strongly Agree that child eats healthy), while adjusting for socio-demographic covariates. All models included survey weights and adjusted for clustering of the data at the city level. Separate models were run for the three food categories. Using the post-estimation margins command, we obtained the average quantities consumed by children in each MyPlate age/sex group, for the three parent perception categories, after controlling for child’s race/ethnicity, mother’s education, household’s poverty level, and household’s participation in WIC and SNAP.

## 3. Results

Parent, child, and household characteristics are summarized in Table 1. Most children were non-Hispanic black (47%) or Hispanic (40%), while white/other made up the remaining 13% of the sample. The majority (68%) of children were from households with incomes below 200% of the Federal Poverty Level (FPL), and 58% had a mother with a high school degree or less. Overall, 54% of parents in our sample strongly agreed their child was eating a healthy diet, compared to 32% somewhat agreeing, and about 14% disagreeing. The majority (59–65%) of parents of younger children (2–3 and 4–8 years old) strongly agreed that their child ate healthy, compared to only 44–50% of parents of older children (9–13 and 14–18 years old) who strongly agreed their child ate healthy (data not shown).

Percentages of children by age/sex categories meeting MyPlate vegetable and fruit, and AHA added sugar recommendations are presented in Table 2. For both vegetables and fruit, fewer older children compared to younger children met the recommendations. For instance, 19% of younger children (2–3 years old) met vegetable recommendations compared to less than 1% of 14–18-year-old males and females. Most 2–3-year-old children met fruit recommendations (94%), compared to only 26–28% of 14–18-year-old males and females. All children in our sample consumed more than 6 teaspoons of added sugars.

**Figure 1:**
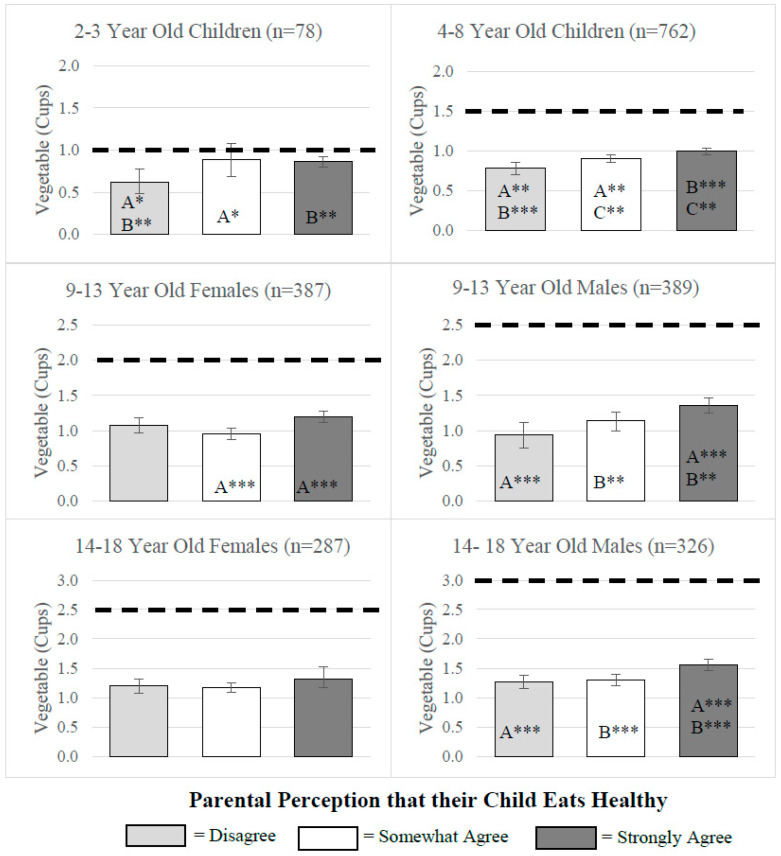
Children’s vegetable consumption across parental perception categories. Notes. Results from multivariate regression analysis showing adjusted average cups of vegetable consumed by parental perception of children’s diet categories. Models included parental perception, sex, age, parental education, federal poverty level, race/ethnicity, SNAP participation, and WIC participation. Bars with the same letter (A, B, or C) represent significant differences in consumption across those perception categories (* *p* ≤ 0.05; ** *p* ≤ 0.01; *** *p* ≤ 0.001). MyPlate recommendations for each age and sex category are represented by black dashed lines.

**Figure 2:**
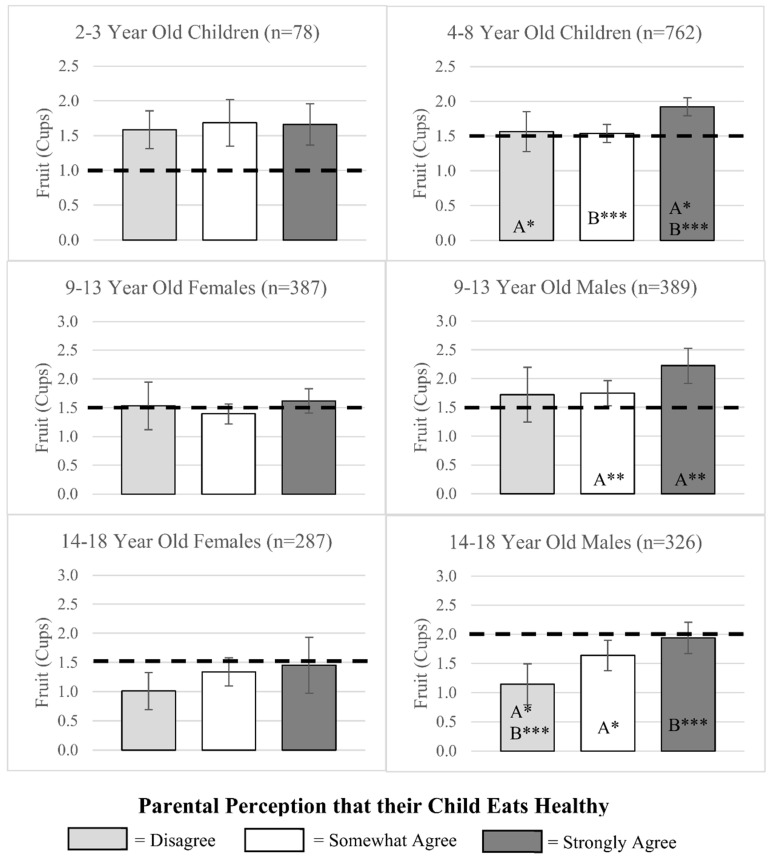
Children’s fruit consumption across parental perception categories. Notes. Results from multivariate regression analysis showing adjusted average cups of fruit consumed by parental perception of children’s diet categories. Models included parental perception, sex, age, parental education, federal poverty level, race/ethnicity, SNAP participation, and WIC participation. Bars with the same letter (A, B, or C) represent significant differences in consumption across those perception categories (* *p* ≤ 0.05; ** *p* ≤ 0.01; *** *p* ≤ 0.001). MyPlate recommendations for each age and sex category are represented by black dashed lines.

**Figure 3:**
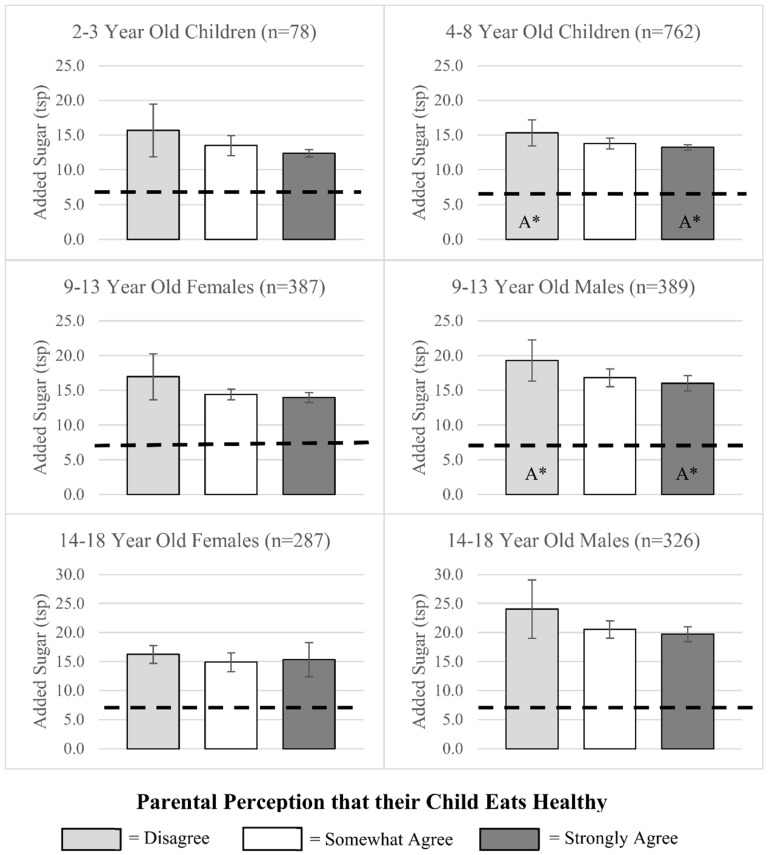
Children’s added sugar consumption across parental perception categories. Notes. Results from multivariate regression analysis showing adjusted average teaspoons of added sugars consumed by parental perception of children’s diet categories. Models included parental perception, sex, age, parental education, federal poverty level, race/ethnicity, SNAP participation, and WIC participation. Bars with the same letter (A, B, or C) represent significant differences in consumption across those perception categories (* *p* ≤ 0.05). American Heart Association recommendations for each age and sex category are represented by black dashed lines.

Results from multivariate regression analyses examining mean consumption by MyPlate age/sex categories across the three levels of parental perception are presented in Figure 1, Figure 2 and Figure 3. Figure 1 and Figure 2 include the MyPlate recommendation for vegetables and fruit, whereas Figure 3 includes the AHA recommendation for added sugars. Average vegetable consumption fell short of recommendations for all age/sex categories (Figure 1). However, in most age/sex categories, significant differences across parental perception categories were observed in amounts of vegetables consumed. For example, all 2-3 year-old children, 4–8-year-old children, 9–13-year-old males and females, and 14–18- year-old males whose parents strongly agreed they ate healthy consumed significantly higher amounts of vegetables compared to those in the same demographic groups with parents who only somewhat agreed or disagreed they ate healthy For older female adolescents (14–18 years), there was no difference in vegetable consumption across parental perception categories.

Among all 4–8-year-old children, 9–13-year-old males, and 14–18-year-old males, fruit consumption varied by parent perception of their child’s diet (Figure 2). In these three age/sex categories, children whose parents strongly agreed that their child ate healthy consumed significantly more fruit compared to children whose parents somewhat agreed or disagreed that their child ate healthy. Notably, no differences in fruit consumption were observed among 9–13 and 14–18-year-old females across parent’s perception categories. With the exception of older adolescents (14–18-year-old males and females), mean fruit consumption was close to or above the recommended levels.

Lastly, Figure 3 shows amounts (teaspoons) of added sugar consumed by children in various age/sex categories by parent perception categories. We found few differences in added sugar consumption by parent perception when examining specific age/sex categories. Only 4–8-year-old children and 9–13-year-old male children of parents who strongly agreed that their child ate healthy consumed significantly less added sugar compared to the same age and sex children of parents who disagreed with the statement. No differences were observed in the amount of sugar consumed by parental perception for any other age/sex group. However, all children, regardless of parental perception, exceeded their recommended intake by consuming more than 6 teaspoons of added sugars.

## 4. Discussion

Across a wide age spectrum (3–18 years), this study is the first to compare parental perception of child’s diet with the child’s consumption of fruits, vegetables, and added sugars, relative to MyPlate and AHA recommendations. While most children, especially in the younger age groups, meet the recommendation for daily amount of fruit, the majority of children in all age/sex categories fell short of the recommendations for vegetables and added sugars, with older children faring worse than younger children. Our results show that, in spite of this sub-optimal consumption, 44–65% of parents strongly agreed their child ate healthy, indicating a mismatch between the healthfulness of their child’s diet and the perceived healthfulness. This observed mismatch occurs despite the fact that children’s dietary behaviors are reported by the parent themselves. Even though parents tend to overestimate the overall healthfulness of their child’s diet, we found an association between children’s eating behaviors and parental perception. This association was observed for several sex/age groups and was in the expected direction, as healthier behaviors corresponded to healthier perceptions. The most marked associations were observed for vegetables, followed by fruits and added sugars.

Consistent with previous studies [5,26], we found that average vegetable consumption was lower than the MyPlate recommendations for all age/sex groups. This lower consumption of vegetables was observed across all parental perception categories. However, children in most age/sex categories (5 out of 6 examined) whose parent strongly agreed they ate healthy consumed more vegetables compared to children with parents in the other two perception categories (somewhat agree and disagree). This suggests that overall, parents seem to be aware that eating vegetables is healthy; however, they might underestimate the optimum quantity of vegetables their child should eat.

With regard to fruit, children (up to 13 years-old) on average tended to consume the recommended amounts, adolescents (age 14–18) did not. This pattern is consistent with nationally representative data [5,27]). When differences in fruit consumption were compared across parental perception categories, they were in the expected direction, showing that consumption was higher for children whose parents strongly agreed that their child ate healthy. This association was observed for 14–18-year old males, but not for 14–18-year-old females. It is therefore likely that parents of adolescent boys may consider fruit as a component of healthy eating more so than parents of adolescent girls. The low consumption of fruit by adolescent girls in our sample and nationally [4], coupled with lack of their parent’s perception that higher fruit consumption is associated with healthy eating is particularly concerning and should be addressed through nutrition education programs.

Lastly, similar to what was observed at the national level [4,7,28], children in our sample, regardless of age and sex, consumed more added sugars than recommended by the AHA. Differences in added sugar intake across parental perception categories were only observed for 4–8-year-old children and 9–13-year-old males. This limited association between sugar consumption and parental perception of children’s diet quality may have two possible explanations. First, parents may not be aware of the overall detrimental effects of added sugars on health and may perceive sugar sweetened foods as an integral part of children’s diets. Second, there is a large selection of highly marketed sugar sweetened beverages that parents may not perceive as unhealthy [29]. Parents have also been shown to consider sugary beverages, such as sports drinks, fruit drinks, and flavored waters, as healthier alternatives to soda [30,31]. National data have shown that although prevalence of soda consumption has decreased, sports/energy drink consumption has tripled among adolescents [32].

Our findings have implications for designing future interventions and studies aimed at improving children’s diets. Although most children fell short of recommendations for vegetables and, to a lesser extent for fruit, higher consumption of these foods was, in most instances, associated with parents’ perception that their child eats healthy. In addition to highlighting the healthful impacts of eating fruits and vegetables, interventions targeting parents should emphasize age appropriate quantities for children to consume. Parents’ inability to associate added sugars with unhealthy diets suggest future interventions should focus on using evidence-based messaging for limiting added sugar consumption, including clear information on sugar content of foods and beverages and negative health consequences of sugar consumption [29].

A major strength of this study was a large, diverse sample of children across a wide age range. Another strength was the use of validated survey questions to assess children’s consumption, with frequency of consumption responses converted into quantities using the established protocol recommended by the NCI. While we recognize that parent reports of their child’s dietary behaviors have some drawbacks [33], using parent reports allowed us to analyze children of different ages (3–18 years-old) and to directly compare child’s dietary behaviors with parental perception of the healthfulness of their child’s diet [20,21]. Parent reports are also subject to social desirability bias, which can result in over-reporting consumption of healthy foods and underreporting unhealthy ones [34]. However, in the current study, social desirability bias would translate into a conservative estimate of the observed mismatch between parental perception and children’s consumption, as parents are likely to have reported a higher consumption of healthy foods like fruits and vegetables and lower consumption of unhealthy beverages and snacks. Future studies should consider using older children’s self-reported diet consumption or other objective consumption measures to assess discrepancies between parental perceptions and children’s diet. The main limitation of the current study was the inclusion of only three food categories, while excluding three of the five MyPlate food group categories (i.e., whole grains, dairy, and lean proteins), as these data were not available in the NJCHS. Lastly, we acknowledge that the construct of an overall healthy diet might be interpreted differently across respondents, as perception questions inherently carry a subjective component.

## 5. Conclusions

Most parents consider their children’s diets to be healthy, even though most children fall short of the recommendations for fruits (except at younger ages) and vegetables and exceed the recommendations for added sugars. Despite some variation across age/sex groups, we found that parents’ perceptions of their children’s diets are positively associated with higher, albeit still inadequate, consumption of fruits and vegetables but show a weak association with consumption of added sugars. Nutrition education efforts aimed at informing parents of the components of a healthy diet should reflect specific age and sex serving amounts that are provided in the DGA and MyPlate resources.

## Figures and Tables

**Table 1:** Demographic characteristics of children in the analytical sample from the New Jersey Child Health Study.

Demographic Variables	*n* = 2229	%
**Sex**		
Female	1086	48.7
**Age**		
2–3 years	78	3.5
4–8 years	762	34.2
9–13 years	776	34.8
14–18 years	613	27.5
**Race/Ethnicity**		
Non-Hispanic White/Other	286	12.8
Non-Hispanic Black	1043	46.8
Hispanic	900	40.4
**Mother’s education**		
Less than High School	421	18.9
High School or Equivalent	876	39.3
Some College	564	25.3
College Degree or More	368	16.5
**Poverty level**		
<200% FPL ^1^	1526	68.5
**WIC ^2^ status**		
Participating	432	19.4
**SNAP ^3^ status**		
Participating	739	33.2
**Parental perception of child’s diet quality as healthy**		
Disagree	311	14.0
Somewhat Agree	711	31.9
Strongly Agree	1207	54.1
**Food categories consumed**	**Mean**	**Std. Deviation**
Fruit (cups)	1.7	1.0
Vegetable (cups)	1.1	0.4
Added Sugars (teaspoons)	15.4	6.2

^1^ FPL: Federal Poverty Level. ^2^ WIC: Special Supplemental Nutrition Program for Women, Infants, and Children. ^3^ SNAP: Supplemental Nutrition Assistance Program.

**Table 2:** Percentage of children meeting MyPlate fruit and vegetable recommendations and the American Heart Association’s (AHA) added sugar limitations.

MyPlate Age/Sex Groups		% Meeting Recommendations		
Category	N	Vegetables	Fruit	Added Sugars
2–3 years (all)	78	19.1	94.7	0.0
4–8 years (all)	762	5.8	50.0	0.0
9–13-year-old females	387	2.2	36.2	0.0
9–13-year-old males	389	9.5	57.2	0.0
14–18-year-old females	287	0.3	28.2	0.0
14–18-year-old males	326	0.5	26.3	0.0
